# Oral Health and Healthy Ageing: A Systematic Review of Longitudinal Studies

**DOI:** 10.3390/dj13070303

**Published:** 2025-07-04

**Authors:** Lujain Sahab, Jonathon Timothy Newton, Wael Sabbah

**Affiliations:** Faculty of Dentistry, Oral & Craniofacial Sciences, King’s College London, London SE5 9RS, UK; lujain.sahab@kcl.ac.uk (L.S.); tim.newton@kcl.ac.uk (J.T.N.)

**Keywords:** healthy aging, oral health, tooth loss, systematic review, longitudinal studies

## Abstract

**Background**: The global rise in life expectancy and the resulting shift toward ageing populations pose significant public health and socioeconomic challenges. As healthy ageing becomes a priority, understanding the factors that support well-being in older age is essential. Oral health is increasingly recognised as a critical determinant of overall health and has been linked to chronic conditions. **Objectives**: To conduct a systematic review of longitudinal studies examining the relationship between oral health and healthy ageing. **Materials and Methods**: Two independent reviewers conducted searches in three databases (MEDLINE, EMBASE, and LILACS) up to April 2025, following a defined search strategy. Grey literature was explored using Open Grey and Google Scholar. The quality and the risk of bias of the included studies were evaluated using the Newcastle Ottawa Quality Assessment Scale (NOS) for longitudinal studies. The review protocol was registered with the International Prospective Register of Systematic Reviews (CRD420251029090). **Results**: Four longitudinal studies reporting the association between oral health and healthy ageing were recognised and included. All selected studies were considered of good quality according to the NOS. The studies varied in defining and measuring healthy ageing, the follow-up period, the sample size, and the measure of oral health; therefore, it was not possible to perform a meta-analysis. The studies included in the review demonstrated a positive relationship between the number of natural teeth and healthy ageing. **Discussion**: Despite variations in the definition of healthy ageing and the application of different oral health indicators, the review identified significant associations between the number of natural teeth and trajectories of healthy ageing. **Conclusions**: This review recognised significant longitudinal associations between oral health measures (number of teeth) and trajectories of healthy ageing. The findings emphasise the need to incorporate oral health into research and policy related to healthy ageing.

## 1. Introduction

The increasing life expectancy and the worldwide trend toward an ageing population pose distinct social and economic challenges for today’s society [1]. Given the global increase in ageing populations, identifying the range of factors that influence healthy ageing has become a significant area of interest in public health and clinical practice. The World Health Organization (WHO) defines healthy ageing as the process of building and sustaining the functional capacity that supports well-being in later life [2].

One factor that has gained considerable attention in the ageing population is oral health. Oral health is vital for general health and well-being, as poor oral health can contribute to systemic conditions like heart disease, diabetes, and respiratory infections [3]. There is a recognised connection between poor oral health and a range of negative complications like pain, infections, and tooth loss, which diminish the ability to consume a varied and nutritious diet [4].

Oral health, especially the retention of natural teeth, contributes in multiple ways to the ageing process. Tooth loss can directly affect nutritional intake due to impaired chewing ability, which may lead to dietary deficiencies that are crucial for maintaining muscle mass, immune function, and cognitive health in older adults [5,6,7]. Additionally, poor oral health is associated with chronic systemic inflammation, largely driven by periodontitis, a common cause of tooth loss, which has been linked to a range of age-related conditions, including cardiovascular disease, diabetes, and cognitive decline [8]. Beyond the physiological impacts, tooth loss may also influence the psychological and social dimensions of ageing. It has been associated with lower self-esteem, reduced social participation, and poorer quality of life, all of which are important components of healthy ageing [9].

Poor oral health imposes a significant economic burden globally through high treatment costs and productivity losses from reduced work capacity and time away from work. Widespread oral diseases, like dental caries and periodontitis, negatively affect employment, earnings, and work performance, with particularly severe consequences for low-income families, who may also face financial strain from caregiving responsibilities. Although spending on dental care varies widely between high- and low-income countries, the economic impact is felt across all regions. Oral diseases contribute to broader social and economic inequalities, as they often reduce earning potential and lead to long-term work limitations, especially among disadvantaged groups. In many low- and middle-income countries, the high cost of treatment results in untreated conditions, deepening the economic burden on individuals and health systems. Despite these widespread effects, a lack of comprehensive data and rigorous research limits our understanding of how these burdens vary across different social and demographic groups [10,11,12].

Despite the availability of cross-sectional studies that suggest associations between oral health status and various indicators of ageing well [13,14], the relationship between oral health and healthy ageing has not been sufficiently explored in a longitudinal context. There is a notable gap in longitudinal evidence examining how changes in oral health status may influence the trajectories of healthy ageing over time. Given the potential impact on enhancing the health and well-being of ageing populations, addressing this gap is critical for understanding causal pathways and informing preventative strategies in older populations. A thorough systematic review is essential to consolidate existing evidence and offer a clearer insight into this relationship.

Thus, this systematic review aims to review and assess longitudinal research examining the connection between oral health and healthy ageing in the older adult population. The main aim of this review is to evaluate the association between oral health (as measured based on any indicator) and the trajectories of healthy ageing over time in older adults.

## 2. Materials and Methods

In line with the Preferred Reporting Items for Systematic Reviews and Meta-analyses (PRISMA) statement guidelines, this systematic review was conducted [15]. The review protocol has been registered in the International Prospective Register of Systematic Reviews (PROSPERO) (Registration number CRD420251029090).

### 2.1. Eligibility Criteria

Eligibility criteria were based on PRISMA guidelines and structured using the PECO framework: P (population) older adults aged 50 and over; E (exposure) oral health; C (comparison) individuals with varying degrees of tooth loss, specifically comparing those with significant tooth loss to those with no or minimal tooth loss; O (outcome) healthy ageing.

### 2.2. Inclusion Criteria

Both retrospective and prospective longitudinal studies were included. The participants consisted of community-dwelling older adults aged 50 years and above. The exposure was defined as any oral health indicator measured at least once during the study period, such as tooth loss, periodontitis, or self-reported oral health. Participants with poor oral health were compared to those with better oral health. Repeated measures of healthy ageing/successful ageing were indicated by any validated scale, measurement, or index (e.g., healthy ageing index, successful ageing index).

### 2.3. Exclusion Criteria

Studies such as intervention trials, cross-sectional research, and those involving children, institutionalised adults, or individuals under 50 years of age were excluded. Also excluded were studies that were non-English, those that did not include an oral health indicator, those that did not examine the link between oral health and healthy ageing, and studies lacking repeated assessments of healthy ageing over time.

The inclusion and exclusion criteria for the studies considered in this review are summarised in Table 1. These criteria are aligned with the PECO framework to ensure a clear and systematic approach to selecting studies based on the population, exposure, comparator, and outcomes.

### 2.4. Information Sources

**Figure 1:**
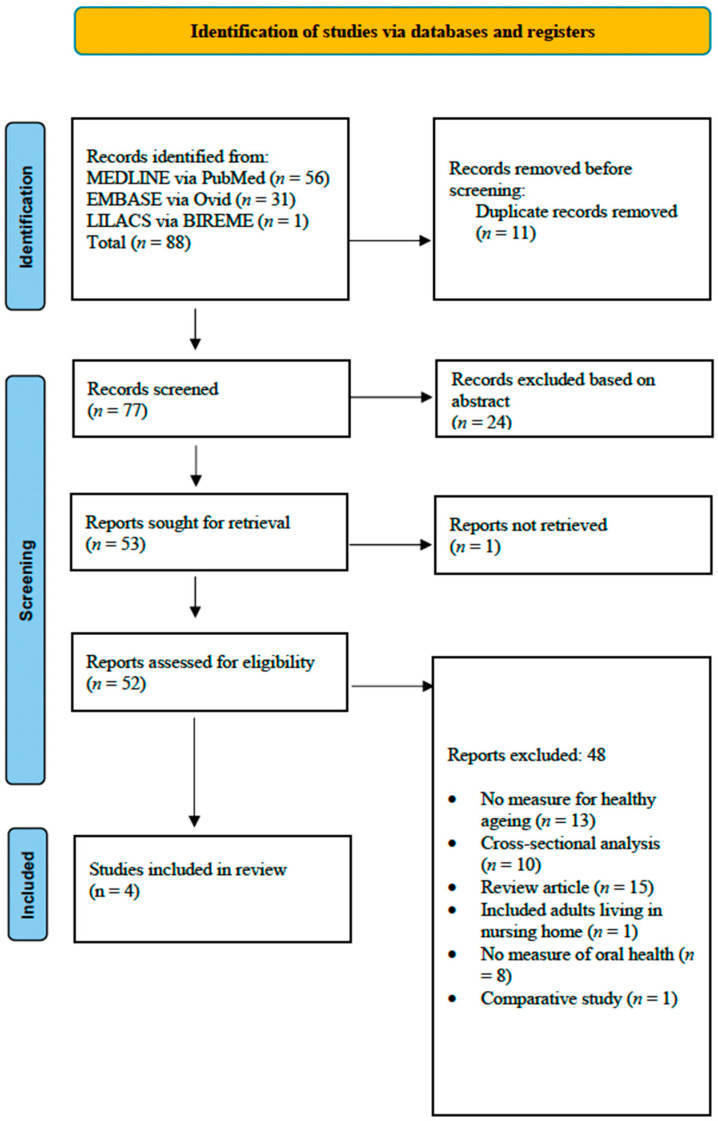
PRISMA flow diagram of selected studies.

Two independent reviewers performed the literature search across three databases: MEDLINE via PubMed, EMBASE via Ovid, and LILACS via BIREME up to April 2025. Additional databases, such as Scopus and Web of Science, were not included due to the substantial overlap in content with MEDLINE and EMBASE. Only published papers were included in the review. To incorporate unpublished studies, the authors reached out to known experts in the field and searched for relevant grey literature on OpenGrey and Google Scholar. Initially, papers were screened for relevance based on their titles and abstracts, with the full text of selected articles being reviewed for final inclusion (Figure 1). Disagreements between the reviewers were resolved through discussion to clarify perspectives based on the predefined criteria. If the disagreement persisted, a third reviewer was consulted to make the final decision. All references identified from the three databases were managed using EndNote 21 software.

The following papers were excluded from the review for specific reasons. Several were review articles and thus not original research [16,17,18,19,20,21,22,23,24,25,26,27,28,29]. Others did not include a measure of oral health [30,31,32,33,34,35,36,37], while some were excluded for being cross-sectional in design [13,14,38,39,40,41,42,43]. Several studies did not include a measure of healthy ageing [44,45,46,47,48,49,50,51,52,53,54,55,56]. One paper was excluded because it was a comparative study [57] and another because it focused on adults living in a nursing home [58].

### 2.5. Search Strategy

Key search terms were determined based on the review question, the inclusion criteria, and a previous systematic review related to the topic. The search used a combination of the following terms across different databases, which identify healthy ageing and oral health concepts: Healthy Ageing OR Healthy Aging OR Ageing Well OR Aging Well OR Successful ageing OR Successful aging AND Oral Health OR Tooth Loss OR Periodontal Diseases OR Dental Caries OR Tooth Decay OR Subjective Oral Health OR Self-reported Oral Health AND Older Adults AND longitudinal AND prospective studies OR Retrospective studies. Date restrictions were for papers published between 1999 to 2025. An English language restriction was applied.

### 2.6. Study Characteristics

Table 2 presents the characteristics of the included studies. The studies focused on adults aged 50 years or older, with follow-up periods varying from four to eleven years. The four studies employed different terms to describe healthy ageing. While the majority used the term ‘healthy ageing’, one study referred to it as ‘successful ageing’. Due to the lack of a universally accepted definition of healthy ageing, various operational definitions have been applied interchangeably across studies [59] with different methods used to measure healthy ageing.

In the field of gerontology, various terms have been employed to describe the concept of “healthy ageing”, including “successful ageing”, “ageing well”, and “active ageing”. While these terms may carry nuanced differences, they fundamentally refer to the same overarching goal: promoting a high quality of life as individuals grow older. Successful ageing often emphasises the achievement of physical and mental health, while ageing well encompasses a broader perspective that includes emotional and social well-being [60,61]. The World Health Organization defines healthy ageing as continued participation in social, economic, cultural, and civic activities, highlighting the intricate and multifaceted nature of the ageing process [62]. Therefore, despite the diversity of terminology, it is essential to recognise that these terms converge on similar principles of health, well-being, and quality of life in later years. This systematic review aims to synthesise findings across these various definitions, highlighting how oral health influences healthy ageing.

**Table 2:** Characteristic of longitudinal studies on oral health and healthy ageing.

Study	Study Design	Country	Data	Population and Setting	Age	Exposure	Outcome
[63]	Longitudinal study (11-year follow-up)	Japan	Ohsaki Cohort, 2006 Study	Baseline = 9947 After exclusions, 8300 participants were included in the analysis	≥65 years	Number of remaining natural teeth that older adults in Japan have at baseline. Participants were categorised based on their number of teeth into four groups: 0–9, 10–19, 20–24, and ≥25 teeth.	Healthy ageing is defined by the presence of all four of the following components: 1—Free of functional disability 2—Free of depression 3—High health-related quality of life (HRQOL) 4—High life satisfaction
[7]	Longitudinal study (10-year follow-up)	United States	The Health and Retirement Study	Baseline = 17,938 Final analysis = 3665	≥50 years at baseline	Total tooth loss individuals were dichotomised into dentate and edentate.	Healthy ageing: assessed as an aggregate measure encompassing three domains: freedom from cognitive impairment, freedom from disability (activities of daily living), and high physical functioning.
[64]	Longitudinal study (9-year follow-up)	Japan	Tsurugaya Project	Baseline = 507 Final follow-up = 450	≥70 years	Number of remaining teeth, assessed by dentists during the 2003 baseline survey. The count excluded retained roots and was classified into three groups: 0–9 teeth, 10–19 teeth, and 20 or more teeth, based on prior research standards.	Maintenance of successful ageing is defined by three criteria: survival, absence of functional disability (as certified by LTCI in Japan), and high health-related quality of life (HRQOL) indicated by an EQ-5D-3L score of 1.000.
[65]	Longitudinal study (4-year follow-up)	China	Chinese Longitudinal Healthy Longevity Survey	Baseline = 2015 participants Final follow-up: 1223	≥65 years	Oral health status: number of natural teeth and denture use. Participants self-reported the number of remaining natural teeth and whether they used dentures. These oral health variables were categorised into four groups based on the number of natural teeth (0, 1–9, 10–19, ≥20) and two categories based on denture use (yes or no).	Healthy ageing: being free of depression, free of ADL (Activities of Daily Living) disability, having high self-rated health (SRH), and high health-related quality of life (HRQOL). Participants were categorised as experiencing healthy ageing if they met these criteria.

### 2.7. Risk of Bias in the Included Studies

According to the Newcastle-Ottawa Scale criteria, Table 3 presents the risk of bias assessment. Two studies received a score of 9 and two scored 8 reflecting good quality. Variations in ratings among the studies were primarily due to differences in the outcome domain scores.

### 2.8. Results of Individual Studies

Across the four studies, a consistent pattern emerges stating that better oral health is positively associated with healthier ageing outcome. Specifically, possessing more natural teeth is positively linked to more favourable healthy ageing outcomes. Although these studies adjusted for various covariates such as age, gender, socioeconomic status, health behaviours, and cognitive function, some limitations were noted, including the reliance on self-reported data and potential biases. Overall, the findings focus on the significance of maintaining oral health as a crucial element in supporting healthy ageing. The association between oral health and the outcome of healthy/successful ageing can be found in Table 4.

### 2.9. Measurement of Healthy Ageing

There was considerable variation in the terminology and measurement used to define healthy ageing. Three studies used the term healthy ageing, while one used successful ageing. While three studies [7,63,65] used the same terminology (healthy ageing), the assessment criteria differed. Details on how each analysis measured the outcome are presented in Table 2.

### 2.10. Oral Health

The four studies reviewed investigated the link between oral health and healthy ageing and reported comparable findings. However, the operational definition of oral health, specifically tooth loss, differed between studies. Details can be found in Table 4.

## 3. Synthesis of the Results

A total of four studies met the eligibility criteria and were incorporated in this systematic review. The small number of included studies reflects the limited and emerging nature of the longitudinal evidence based on the association between oral health and healthy ageing in older adults. Due to the variability in study designs, outcome measures, and oral health indicators across the included studies, conducting a meta-analysis was not possible. Instead, a narrative synthesis was performed, in line with The Economic and Social Research Council’s guidelines for improving transparency and rigor in systematic reviews [66]. This process involved four main steps: creating a theoretical model, conducting an initial synthesis, examining relationships between studies, and evaluating the reliability of the findings

A preliminary conceptual framework was established to guide the synthesis. It was hypothesised that improved oral health, particularly the number of remaining teeth, denture use, and being dentate, is associated with healthier ageing outcomes. This relationship is theorised consistently with the WHO framework on healthy ageing [2].

The data from the selected studies were gathered and tabulated to facilitate a comparison of study characteristics, outcome measures, and results (Table 2).

Studies were grouped based on the dependent variable, predictor variable, and measure of association and covariates. The predictor variable, in this case, oral health indicators, were assessed using one or more of the following: denture use, number of remaining teeth, and edentulism.

Across the included studies, healthy ageing was measured using multidimensional criteria, reflecting the complex and varied ways it is conceptualised in the literature. Although the definitions differed slightly, all studies incorporated elements of physical functioning, psychological well-being, and quality of life. Despite variations in specific indicators, the definitions consistently centred on independence, well-being, and quality of life, allowing for a coherent synthesis of findings while acknowledging differences in operationalisation. A description of the predictor variable can be found in Table 2.

Across the studies, a general trend was observed that a greater number of remaining natural teeth was positively associated with healthy ageing. Matsuyama, Lu [63], Jilili and Cheng [65], and Tanji and Komiyama [64] all reported significantly increased odds for healthy ageing among individuals with ≥20 natural teeth, even after adjusting for covariates such as age, sex, education, and health behaviours. Conversely, Sahab and Newton [7] identified edentulism as a significant predictor of poorer ageing outcomes.

Studies with extended follow-up periods typically offered more robust evidence of a link between oral health and healthy ageing. Although the covariates varied between studies, the association between oral health and healthy ageing remained robust across different populations and study designs. Despite these consistencies, certain studies depended on self-reported oral health data, which could lead to recall bias

The quality of the included studies was evaluated using the Newcastle-Ottawa Scale (NOS) designed for longitudinal studies, and all were rated as good quality. However, the differences in study methodologies and descriptions of healthy ageing presented limitations to direct comparisons. Despite these differences, the consistency of associations across diverse settings supports the credibility of the findings.

## 4. Discussion

Given the global rise in ageing populations and evolving demographic trends, understanding the key determinants of healthy ageing is crucial for informing targeted public health strategies. Oral health has become a critical yet often underrecognised factor influencing the ageing process. Age-related changes in dentition, such as enamel thinning, increased tooth wear, and reduced salivation, are common in older adults, making them more susceptible to dental issues like tooth sensitivity and caries [67]. Bruxism, or teeth grinding, further exacerbates these changes by accelerating tooth wear, causing fractures, and contributing to temporomandibular disorders, particularly in individuals with underlying health conditions [68,69]. These factors underscore the importance of early diagnosis and management to maintain oral health in older populations.

The findings of this review highlight that oral health indicators such as the number of remaining teeth, presence of tooth loss, and denture use play a significant role in shaping healthy ageing outcomes.

To the extent of our understanding, no other systematic review has solely focused on longitudinal studies investigating the relationship between oral health, particularly the number of natural teeth, and the trajectories of healthy ageing.

### 4.1. Key Findings from the Review—Summary of Evidence

Four longitudinal studies were identified in this systematic review. The studies took place in Japan [63,64], the United States [7], and China [65].

According to the NOS, each study was evaluated and deemed to have a low risk of bias. All included papers showed high heterogeneity in terminology, definitions, and measurements of dependent and outcome variables. The covariates that were selected and tested also varied. Furthermore, there were differences in the follow-up durations, sample sizes, and populations. These variations across the studies made it difficult to combine the results, preventing the possibility of conducting a meta-analysis.

All papers provided justification for attrition. Reasons included participants being excluded due to moving out of the study area, death or incident disability, missing data, and incomplete responses.

Since age is a key determinant of healthy ageing, the duration of follow-up periods is essential for tracking the trajectories of healthy ageing. The longest follow-up in the review spanned 11 years [63], while the shortest was 4 years [65]. Extended follow-up periods enhance the reliability of outcome measurements and provide a more rigorous evaluation of the association under investigation. Furthermore, all the studies differed in sample sizes at the final follow-up. The largest included 8300 participants [63], and the smallest included 450 participants [64].

The included studies gave evidence of a longitudinal relationship between oral health (specifically number of remaining natural teeth) and healthy ageing. Even after accounting for behavioural factors, such as alcohol intake, tobacco use, and socioeconomic factors, the association persisted.

The assessment of the outcome varied between studies; all definitions are presented in Table 2. While different definitions were used for healthy/successful ageing in the literature, this review was not limited to a specific definition to include a greater number of studies that demonstrated transitions or trajectories of healthy/successful ageing.

The association between tooth loss and adverse health outcomes can be explained through several interconnected mechanisms, biological, behavioural, and social. Matsuyama, Lu [63] concluded that having enough natural teeth helps maintain chewing functions, enabling pleasurable eating and better nutrition, as well as supporting mental and social well-being. Sahab and Newton [7] found that tooth loss significantly impacts nutritional intake, which in turn influences healthy ageing in older American adults. Socioeconomic factors and health behaviours like smoking and physical activity also play crucial roles in this relationship. Tanji and Komiyama [64] showed that biological factors like oral inflammation and the nutritional status partially mediate the relationship between the number of remaining teeth and successful ageing, as tooth loss can lead to systemic inflammation and impaired nutrition. Social aspects, particularly mental health, play a crucial role, with psychological well-being substantially influencing the link between tooth retention and the maintenance of successful ageing. The results of Jilili and Cheng [65] indicate that biologically, tooth loss leads to difficulties in chewing and swallowing, resulting in poor nutrition and malnutrition, which negatively impact health; behaviourally and socially, tooth loss may reduce social participation and communication, with both essential for healthy ageing.

### 4.2. Latest Work on the Topic

This review highlights the significant impact of tooth loss on general health, particularly in older adults. Studies have shown that tooth loss is not only an oral health concern but also a broader issue with implications for systemic health [70,71,72] suggesting that maintaining natural dentition may help preserve general health. These findings underscore the importance of preserving natural teeth as a preventive strategy, not only for maintaining oral health but also for promoting overall well-being and preventing chronic conditions associated with tooth loss. Given the growing evidence of the broader implications of tooth loss, future research should focus on understanding the underlying mechanisms and developing effective interventions to prevent or mitigate its effects on general health.

### 4.3. Mechanism of Action

Integrating oral health into healthy ageing frameworks is increasingly recognised as essential for promoting the overall well-being of older adults. Oral health affects multiple dimensions of ageing, including nutrition, cognitive function, social participation, and systemic disease risk. Briguglio and Wainwright [72] highlight the value of a multidisciplinary approach to oral health care, especially in clinical settings such as pre- and post-operative management for older adults, where the oral health status can influence surgical outcomes and recovery. At the policy level, incorporating oral health assessments into primary care systems can help identify risks early and promote preventive care. The FDI World Dental Federation (2023) advocates for the systematic inclusion of oral health in national ageing and public health strategies, suggesting actions such as training healthcare professionals in oral health screening and integrating dental care into routine geriatric services. In addition, addressing the broader social determinants of oral health—such as income, education, and access to care—is vital. Patel and Gallagher [21] emphasise that an integrated, life-course approach is needed to respond to the oral health needs of ageing populations, especially as poor oral health continues to disproportionately affect vulnerable groups. By embedding oral health within healthy ageing policies, both individual outcomes and healthcare system sustainability can be improved through the prevention of avoidable disease and enhanced quality of life in later years.

### 4.4. Limitations

Several limitations were identified within the studies included in the review. The small number of included studies (*n* = 4) limits the generalisability of the findings and the strength of the conclusions. However, this also highlights the importance of the review, as it underscores the scarcity of longitudinal studies examining the relationship between oral health and healthy ageing, an area that remains underexplored and in need of further research. There were variations in the measurement of the dependent variable, measurement of the outcome, the included covariates, and follow-up periods. Due to these inconsistencies, it was not feasible to merge the findings, making a meta-analysis unachievable. Three studies [7,64,65] utilised self-reported data, which poses a risk of recall bias and could affect the accuracy and validity of their individual findings. A potential limitation is the inclusion of the authors’ own study in the analysis. To mitigate potential bias, the authors adhered strictly to predefined eligibility criteria and used established risk-of-bias assessment tools to evaluate all studies. Although grey literature was searched and authors were contacted, unpublished research could not be identified. Therefore, there remains a risk of publication bias, since studies reporting negative findings are often not made publicly available.

## 5. Conclusions

The review found strong long-term links between oral health, specifically having a greater number of natural teeth, and trajectories of healthy ageing. This positive relationship highlights the importance of oral health as a key aspect of general well-being, especially in older adulthood. Importantly, most studies found that this association remained robust even after adjusting for a range of confounding factors, highlighting its potential independent impact on ageing outcomes. These findings support the growing call to integrate oral health into broader public health strategies and ageing policies, recognising that maintaining natural teeth may contribute not only to physical health but also to social participation, nutrition, and quality of life as people age. To improve future research and enable better comparisons across studies, it is essential to adopt more consistent and standardised definitions of both healthy ageing and oral health.

## Figures and Tables

**Table 1:** Inclusion and exclusion criteria aligned with the PECO framework.

PECO Framework	Inclusion Criteria	Exclusion Criteria
P—population	Community-dwelling older adults aged 50 years and above.	Children, institutionalised adults, or individuals under 50 years of age.
E—exposure	Oral health indicators (e.g., tooth loss, periodontitis, or self-reported oral health), measured at least once during the study period.	Studies that do not include an oral health indicator or examine the link between oral health and healthy ageing.
C—comparison	Participants with poor oral health compared to those with better oral health.	Studies without a comparator group or those not comparing different oral health statuses.
O—outcome	Healthy ageing/successful ageing, measured based on validated scales, indices, or measurements (e.g., healthy ageing index, successful ageing index).	Studies lacking repeated assessments of healthy ageing over time or those that did not use a valid scale or measurement.

**Table 3:** Methodological assessment of included studies using the Newcastle-Ottawa Scale (NOS) for longitudinal studies.

Study	Study Design	Selection				Comparability	Outcome			Overall Score and Quality
		Representativeness of the Sample	Selection of the Non-Exposed Cohort	Ascertainment of Exposure	Demonstration That the Outcome of Interest Was Not Present at the Start of the Study	Based on Design and Analysis	Assessment of the Outcome	Was the Follow-Up Long Enough for Outcomes to Occur	Adequacy of Follow-Up	
[63]	Longitudinal	*	*	*	*	**	*	*	*	9 Good Quality
[7]	Longitudinal	*	*	*	*	**	*	*		8 Good Quality
[64]	Longitudinal	*	*	*	*	**	*	*	*	9 Good Quality
[65]	Longitudinal	*	*	*	*	**	*		*	8 Good Quality

* and ** studies meeting risk of bias criteria.

**Table 4:** Association between oral health and healthy ageing.

Study	Dependent Variable	Predictor	Description of the Predictor	Adjusted Measure of Association (95% CI)	Covariates	Results	Comments
[63]	Healthy ageing	Number of teeth	Number of remaining teeth, categorised into four groups: 0–9 teeth 10–19 teeth 20–24 teeth ≥25 teeth	Odds ratio (OR) 95% CI. Regarding the association between the number of remaining teeth and healthy ageing, the multivariate-adjusted ORs compared to the reference group (0–9 teeth) were as follows: 10–19 teeth: OR = 0.98 (95% CI: 0.77–1.26) 20–24 teeth: OR = 1.28 (95% CI: 1.01–1.63) ≥25 teeth: OR = 1.59 (95% CI: 1.24–2.03).	Age, gender, smoking status, drinking status, time spent walking per day, sleep duration, education level, history of disease, cognitive function score, and social participation	A greater number of remaining teeth is associated with a higher likelihood of healthy ageing and increased survival probability, independent of other health and demographic factors.	The reliance on self-reported data for the number of teeth may introduce misclassification bias.
[7]	Healthy Ageing	Oral health: tooth loss and nutrition	Complete tooth loss: a binary variable indicating whether participants have teeth (dentate) or are edentate (without teeth).	The adjusted measure of association between tooth loss (edentulism in 2012) and healthy ageing in 2016 was −0.15 with a 95% confidence interval of (−0.15, −0.14).	Age, gender, ethnicity (race), education level, wealth, income, marital status, smoking, physical activities, BMI	Tooth loss (edentulism) was significantly associated with poorer healthy ageing.	The study acknowledges several limitations, notably the timeframe of the data used, which may restrict the ability to account for recent societal shifts, lifestyle changes, or health events.
[64]	Maintaining successful ageing	Number of remaining teeth at baseline.	Number of teeth categorised into 3 groups: 0–9 teeth 10–19 teeth ≥20 teeth	The adjusted associations between the number of remaining teeth and the preservation of successful ageing are reported as prevalence ratios (PRs) with 95% confidence intervals (CIs). Participants with 10–19 teeth had a PR of 1.39 (95% CI: 0.81–2.36) compared to those with 0–9 teeth. Participants with ≥20 teeth had a PR of 1.58 (95% CI: 1.002–2.50) compared to those with 0–9 teeth.	Sex, Age, Medical history, smoking status, drinking status, metabolic equivalent/day score reflecting physical activity levels, education level, Mini-Mental State Examination score, C-reactive protein levels (for inflammation), body mass index, serum albumin levels (reflecting nutritional status)	There was a significant association between the number of remaining teeth and the maintenance of successful ageing. Participants with twenty or more teeth had a higher prevalence ratio (PR) for maintaining successful ageing compared to those with 0–9 teeth, even after an adjustment for multiple covariates. Specifically, the fully adjusted model showed that individuals with ≥20 teeth were 58% more likely to maintain successful ageing compared to those with fewer teeth.	The study primarily relied on baseline assessments of the number of remaining teeth without accounting for changes in oral health status or tooth loss that could have occurred during the follow-up period, which might influence the association with successful ageing.
[65]	Healthy ageing	(1) Number of natural teeth (2) Denture use	Oral status Number of natural teeth: categorised into 4 groups: 0 teeth, 1–9 teeth, 10–19 teeth, and ≥20 teeth. Denture use: Dichotomised: (1) With dentures (2) Without dentures.	When examining the number of natural teeth, having ≥20 teeth was associated with higher odds of healthy ageing, with an adjusted OR of 2.09 (95% CI: 1.54–2.84) compared to those with no natural teeth.	Gender, age, marital status, financial strain, smoking, and social engagement	Older adults in China with 20 or more natural teeth have a markedly higher likelihood of experiencing healthy ageing compared to those with fewer or none. The adjusted odds ratio was 2.09 (95% CI: 1.54–2.84).	The follow-up period was relatively short, spanning only four years, Self-reported measures for tooth loss and denture use could introduce reporting bias.

## Data Availability

Not applicable.

## References

[1] Cylus J., Roubal T., Ong P., Barber S., Sagan A., Normand C., Figueras J., North J., White C. (2019). European Observatory Policy Briefs. Sustainable Health Financing with an Ageing Population: Implications of Different Revenue Raising Mechanisms and Policy Options.

[2] WHO (2015). World Report on Ageing and Health.

[3] WHO (2024). Ageing and Health. https://www.who.int/news-room/fact-sheets/detail/ageing-and-health.

[4] Chan A.K.Y., Tsang Y.C., Jiang C.M., Leung K.C.M., Lo E.C.M., Chu C.H. (2023). Diet, Nutrition, and Oral Health in Older Adults: A Review of the Literature. Dent. J..

[5] Nowjack-Raymer R.E., Sheiham A. (2007). Numbers of natural teeth, diet, and nutritional status in US adults. J. Dent. Res..

[6] Yoshida M., Kikutani T., Yoshikawa M., Tsuga K., Kimura M., Akagawa Y. (2011). Correlation between dental and nutritional status in community-dwelling elderly Japanese. Geriatr. Gerontol. Int..

[7] Sahab L., Newton J.T., Sabbah W. (2025). The Nutritional Pathway Between Tooth Loss and Healthy Ageing: A Longitudinal Study of Older American Adults. Nutrients.

[8] Hajishengallis G. (2015). Periodontitis: From microbial immune subversion to systemic inflammation. Nat. Rev. Immunol..

[9] Bernabé E., de Oliveira C., de Oliveira Duarte Y.A., Bof de Andrade F., Sabbah W. (2023). Social participation and tooth loss, vision, and hearing impairments among older Brazilian adults. J. Am. Geriatr. Soc..

[10] Jevdjevic M., Listl S. (2025). Global, Regional, and Country-Level Economic Impacts of Oral Conditions in 2019. J. Dent. Res..

[11] Herndon J.B., Rubin M.S., Reusch C., Edelstein B.L. (2024). A scoping review of the economic impact of family oral health: Implications for public health, research, and policy. J. Public Health Dent..

[12] Peres M.A., Macpherson L.M.D., Weyant R.J., Daly B., Venturelli R., Mathur M.R., Listl S., Celeste R.K., Guarnizo-Herreño C.C., Kearns C. (2019). Oral diseases: A global public health challenge. Lancet.

[13] Newman-Norlund R.D., Kudaravalli S., Merchant A.T., Fridriksson J., Rorden C. (2024). Exploring the link between tooth loss, cognitive function, and brain wellness in the context of healthy aging. J. Periodontal Res..

[14] Barboza-Solís C., Porras-Chaverri M., Fantin R. (2019). Is tooth loss important when evaluating perceived general health? Findings from a nationally representative study of Costa Rican adults. Community Dent. Oral Epidemiol..

[15] Page M.J., McKenzie J.E., Bossuyt P.M., Boutron I., Hoffmann T.C., Mulrow C.D., Shamseer L., Tetzlaff J.M., Akl E.A., Brennan S.E. (2021). The PRISMA 2020 statement: An updated guideline for reporting systematic reviews. BMJ.

[16] Poser M., Sing K.E.A., Ebert T., Ziebolz D., Schmalz G. (2023). The rosetta stone of successful ageing: Does oral health have a role?. Biogerontology.

[17] Friedman P.K., Lamster I.B. (2016). Tooth loss as a predictor of shortened longevity: Exploring the hypothesis. Periodontol 2000.

[18] Klionsky D.J., Abdel-Aziz A.K., Abdelfatah S., Abdellatif M., Abdoli A., Abel S., Abeliovich H., Abildgaard M.H., Abudu Y.P., Acevedo-Arozena A. (2021). Guidelines for the use and interpretation of assays for monitoring autophagy (4th edition)(1). Autophagy.

[19] Cecchin-Albertoni C., Deny O., Planat-Bénard V., Guissard C., Paupert J., Vaysse F., Marty M., Casteilla L., Monsarrat P., Kémoun P. (2024). The oral organ: A new vision of the mouth as a whole for a gerophysiological approach to healthy aging. Ageing Res. Rev..

[20] Weintraub J.A., Kaeberlein M., Perissinotto C., Atchison K.A., Chen X., D’Souza R.N., Feine J.S., Ghezzi E.M., Kirkwood K.L., Ryder M. (2023). Geroscience: Aging and Oral Health Research. Adv. Dent. Res..

[21] Patel R., Gallagher J.E. (2024). Healthy ageing and oral health: Priority, policy and public health. BDJ Open.

[22] Chan A.K.Y., Chu C.H., Ogawa H., Lai E.H. (2024). Improving oral health of older adults for healthy ageing. J. Dent. Sci..

[23] Müller F., Shimazaki Y., Kahabuka F., Schimmel M. (2017). Oral health for an ageing population: The importance of a natural dentition in older adults. Int. Dent. J..

[24] Patel J., Wallace J., Doshi M., Gadanya M., Ben Yahya I., Roseman J., Srisilapanan P. (2021). Oral health for healthy ageing. Lancet Healthy Longev..

[25] Fukai K., Dartevelle S., Jones J. (2022). Oral Health for Healthy Ageing: A People-centred and Function-focused Approach. Int. Dent. J..

[26] Fukai K., Ogawa H., Hescot P. (2017). Oral health for healthy longevity in an ageing society: Maintaining momentum and moving forward. Int. Dent. J..

[27] Bassim C.W. (2018). Oral Health in Healthy Aging. J. Am. Geriatr. Soc..

[28] Kiyak H.A. (2000). Successful aging: Implications for oral health. J. Public Health Dent..

[29] Arroyo-Quiroz C., Brunauer R., Alavez S. (2020). Factors associated with healthy aging in septuagenarian and nonagenarian Mexican adults. Maturitas.

[30] Jang H.Y. (2020). Factors Associated with Successful Aging among Community-Dwelling Older Adults Based on Ecological System Model. Int. J. Environ. Res. Public Health.

[31] Cha N.H., Seo E.J., Sok S.R. (2012). Factors influencing the successful aging of older Korean adults. Contemp. Nurse.

[32] Ferdows N.B., Jensen G.A., Tarraf W. (2018). Healthy Aging After Age 65: A Life-Span Health Production Function Approach. Res. Aging.

[33] Wang X., Xie J., Shang M., Yin P., Gu J. (2024). Healthy aging trajectories and their predictors among Chinese older adults: Evidence from a 7-year nationwide prospective cohort study. Arch. Gerontol. Geriatr..

[34] Hsu H.C., Jones B.L. (2012). Multiple trajectories of successful aging of older and younger cohorts. Gerontologist.

[35] Chong M.S., Tan C.N., Yew S., Lim J.P., Lim W.S., Lieu P.K. (2018). Successful Ageing in Nonagenarians: Bio-psychosocial Factors Determining Successful Ageing in Long-Lived Older Adults. J. Am. Med. Dir. Assoc..

[36] Subramaniam M., Abdin E., Vaingankar J.A., Sambasivam R., Seow E., Picco L., Chua H.C., Mahendran R., Ng L.L., Chong S.A. (2019). Successful ageing in Singapore: Prevalence and correlates from a national survey of older adults. Singap. Med. J..

[37] Kim E.-K., Lee S.K., Choi Y.-H., Tanaka M., Hirotsu K., Kim H.C., Lee H.-K., Jung Y.-S., Amano A. (2017). Relationship between chewing ability and cognitive impairment in the rural elderly. Arch. Gerontol. Geriatr..

[38] Delbari A., Ghavidel F., Rashedi V., Bidkhori M., Saatchi M., Hooshmand E. (2024). Evaluation of oral health status in the population above 50: Evidence from the ardakan cohort study on aging (ACSA). BMC Oral Health.

[39] Ng T.P., Broekman B.F., Niti M., Gwee X., Kua E.H. (2009). Determinants of successful aging using a multidimensional definition among Chinese elderly in Singapore. Am. J. Geriatr. Psychiatry.

[40] Sert E.N., Ilgaz A. (2025). Successful Aging and Self-Neglect Among Community-Dwelling Older People. Public Health Nurs..

[41] Li C.I., Lin C.H., Lin W.Y., Liu C.S., Chang C.K., Meng N.H., Lee Y.D., Li T.C., Lin C.C. (2014). Successful aging defined by health-related quality of life and its determinants in community-dwelling elders. BMC Public Health.

[42] Rivadeneira M.F., Mendieta M.J., Villavicencio J., Caicedo-Gallardo J., Buendía P. (2021). A multidimensional model of healthy ageing: Proposal and evaluation of determinants based on a population survey in Ecuador. BMC Geriatr..

[43] Tan L.F., Chan Y.H., Merchant R.A. (2022). Association between dentition and frailty and cognitive function in community-dwelling older adults. BMC Geriatr..

[44] Kiuchi S., Cooray U., Aida J., Osaka K., Chan A., Malhotra R., Peres M.A. (2023). Effect of Tooth Loss on Cognitive Function among Older Adults in Singapore. J. Dent. Res..

[45] Gil-Montoya J.A., de Mello A.L., Barrios R., Gonzalez-Moles M.A., Bravo M. (2015). Oral health in the elderly patient and its impact on general well-being: A nonsystematic review. Clin. Interv. Aging.

[46] Dintica C.S., Rizzuto D., Marseglia A., Kalpouzos G., Welmer A.K., Wårdh I., Bäckman L., Xu W. (2018). Tooth loss is associated with accelerated cognitive decline and volumetric brain differences: A population-based study. Neurobiol. Aging.

[47] Lu X.Z., Sun Y., Chen X. (2023). Research progress in the association between tooth loss and cognitive decline in the elderly population. Zhonghua Kou Qiang Yi Xue Za Zhi.

[48] Taraghi Z., Fanni-Saberi L., Yazdani-Charati J., Meskini L. (2024). The Relationship Between Oral Health and Cognitive Status of the Elderly. Iran. Red Crescent Med. J. (IRCMJ).

[49] Naah F.L., Njong A.M., Kimengsi J.N. (2020). Determinants of Active and Healthy Ageing in Sub-Saharan Africa: Evidence from Cameroon. Int. J. Environ. Res. Public Health.

[50] Zhang W., Wu Y.Y., Wu B. (2018). Does Oral Health Predict Functional Status in Late Life? Findings From a National Sample. J. Aging Health.

[51] Chen X., Douglas C.E., Preisser J.S., Naorungroj S., Beck J.D. (2018). Oral health trajectories in community-dwelling older adults in the last 3 years of life. Spec. Care Dent..

[52] Rouxel P., Tsakos G., Chandola T., Watt R.G. (2018). Oral Health-A Neglected Aspect of Subjective Well-Being in Later Life. J. Gerontol. B Psychol. Sci. Soc. Sci..

[53] Bassim C.W., MacEntee M.I., Nazmul S., Bedard C., Liu S., Ma J., Griffith L.E., Raina P. (2020). Self-reported oral health at baseline of the Canadian Longitudinal Study on Aging. Community Dent. Oral Epidemiol..

[54] Johansson A.K., Omar R., Unell L., Sannevik J., Mastrovito B., Carlsson G.E., Johansson A. (2020). Changes in conditions related to reported oral and general health over a ten-year period as reflected in two cohorts of 75-year-old subjects examined in 2007 and 2017. J. Oral Rehabil..

[55] Kang J., Wu B., Bunce D., Ide M., Pavitt S., Wu J. (2019). Cognitive function and oral health among ageing adults. Community Dent. Oral Epidemiol..

[56] Steele J.G., Sanders A.E., Slade G.D., Allen P.F., Lahti S., Nuttall N., Spencer A.J. (2004). How do age and tooth loss affect oral health impacts and quality of life? A study comparing two national samples. Community Dent. Oral Epidemiol..

[57] Duman İ., Kazak Salti A., Vefikuluçay Yilmaz D. (2025). Factors Affecting Successful Aging of Older Adults and the Relationship Between Leisure Activities Duration and Successful Aging. J. Appl. Gerontol..

[58] Urtamo A., Jyväkorpi S.K., Strandberg T.E. (2019). Definitions of successful ageing: A brief review of a multidimensional concept. Acta Biomed..

[59] Rowe J.W., Kahn R.L. (1997). Successful aging. Gerontologist.

[60] Bowling A. (2008). Enhancing later life: How older people perceive active ageing?. Aging Ment. Health.

[61] WHO (2002). Active Ageing: A Policy Framework.

[62] Matsuyama S., Lu Y., Aida J., Tanji F., Tsuji I. (2022). Association between number of remaining teeth and healthy aging in Japanese older people: The Ohsaki Cohort 2006 Study. Geriatr. Gerontol. Int..

[63] Tanji F., Komiyama T., Ohi T., Hattori Y., Watanabe M., Lu Y., Tsuji I. (2020). The Association between Number of Remaining Teeth and Maintenance of Successful Aging in Japanese Older People: A 9-Year Longitudinal Study. Tohoku J. Exp. Med..

[64] Jilili M., Cheng Y. (2024). The relationship between oral status and healthy aging in Chinese older adults: A community cohort study. J. Public Health Dent..

[65] Popay J., Roberts H., Sowden A., Petticrew M., Arai L., Rodgers M., Britten N., Roen K., Duffy S. (2006). Guidance on the Conduct of Narrative Synthesis in Systematic Reviews: A Product from the ESRC Methods Programme.

[66] Müller F., Naharro M., Carlsson G.E. (2007). What are the prevalence and incidence of tooth loss in the adult and elderly population in Europe?. Clin. Oral Implant. Res..

[67] Lobbezoo F., Ahlberg J., Glaros A.G., Kato T., Koyano K., Lavigne G.J., de Leeuw R., Manfredini D., Svensson P., Winocur E. (2013). Bruxism defined and graded: An international consensus. J. Oral Rehabil..

[68] Zieliński G., Pająk A., Wójcicki M. (2024). Global Prevalence of Sleep Bruxism and Awake Bruxism in Pediatric and Adult Populations: A Systematic Review and Meta-Analysis. J. Clin. Med..

[69] Li L., Zhang Q., Yang D., Yang S., Zhao Y., Jiang M., Wang X., Zhao L., Liu Q., Lu Z. (2023). Tooth loss and the risk of cognitive decline and dementia: A meta-analysis of cohort studies. Front. Neurol..

[70] Qian Y., Cai B., Chi F., Yao C., Zhang L., Qi L., Jiang Y., Wang X. (2023). Alveolar bone loss and tooth loss contribute to increase in cancer mortality among older patients. BMC Oral Health.

[71] Dong L., Ji Z., Hu J., Jiang Q., Wei W. (2025). Oral microbiota shifts following tooth loss affect gut health. BMC Oral Health.

[72] Briguglio M., Wainwright T.W., Latella M., Ninfa A., Cordani C., Colombo C., Banfi G., Francetti L., Corbella S. (2024). A Proposal for a Multidisciplinary Integrated Oral Health Network for Patients Undergoing Major Orthopaedic Surgery (IOHN-OS). Geriatrics.

